# HPV Prevalence and Genotype Distribution Among Women From Hengyang District of Hunan Province, China

**DOI:** 10.3389/fpubh.2021.710209

**Published:** 2021-11-05

**Authors:** Shuang-yang Tang, Ya-qi Liao, Yu Hu, Hai-yan Shen, Yan-ping Wan, Yi-mou Wu

**Affiliations:** Institute of Pathogenic Biology, School of Basic Medicine Sciences, Hengyang Medical School, University of South China, Hengyang, China

**Keywords:** human papillomavirus, prevalence, genotype, distribution, cervical cancer

## Abstract

Most cervical cancers were closely associated with human papillomavirus (HPV) infections. Therefore, understanding the ecological diversity of HPV prevalence and genotype distribution among various populations in different geographical regions was essential for optimizing HPV vaccination and maximizing the vaccination effects. A total of 12,053 patient data from the three-level hospitals in Hengyang city were retrospectively analyzed. In this study, the HPV prevalence was 10.16% overall, and the multiple-type infection rate was 1.83%. The HR-HPV infection rate was 8.52%. The top six HPV genotypes were as follows in descending order: HPV16, HPV58, HPV52, HPV39, HPV51, and HPV53. The HPV prevalence in the group above 60 years old was the most, and their HR-HPV infection rate corresponded to the most too. The infection rates of HPV and HR-HPV among outpatients were both lower than those among the hospitalized-patients, respectively. Among the hospitalized-patients, the infection rates of HPV and HR-HPV among the 50–60 years group were the most in both. The HR-HPV ratio-in-positive among HPV-positive patients with the histopathologic examination was higher than that among those patients without. Among 52 HPV-positive patients with cervical squamous carcinoma, the ratio-in-positive of HPV16 was 61.54%. This study demonstrated that the HPV prevalence varied with age among women from Hengyang district of Hunan province in China and showed that HPV16, HPV58, HPV52, HPV39, HPV51, and HPV53 genotypes were more popularly distributed in this region, which could provide the experimental basis for Chinese public health measures on cervical cancer prevention.

## Introduction

Human papillomavirus (HPV) is a spherically non-enveloped and double-stranded DNA virus and prefers to infect the epithelial cells of the skin and mucous membrane. HPV has more than 200 genotypes, and its infection is widespread in the crowd (1). According to the disease risk caused by HPVs, they were divided into low-risk HPV (LR-HPV) genotype and high-risk HPV (HR-HPV) genotype. Nearly all cervical cancers and also a large proportion of epithelial cancers including some vulvar, vaginal, anal, penile, and oropharyngeal cancers were caused by HPVs (2). Cervical cancer had become the second leading cause of women's death globally, especially in developing countries according to the reports of the International Agency for Cancer Research (3, 4). In China, there were more and more new cases of cervical cancer (5, 6), suggesting that cervical cancer had put a heavy burden on Chinese women, where HPV-related infections were the primary cause of their morbidity and mortality (3). At present, the potential long-term solution for preventing cervical cancer in developing countries is vaccination against HPV. The bivalent HPV vaccine (Cervarix) against HPV16/18, the tetravalent vaccine (Gardasil) against HPV6/11/16/18, and the 9-valent HPV vaccine (2nd Gardasil) against HPV 6/11/16/18/31/33/45/52/58 can be possible to prevent most cervical cancers related their corresponding HPV genotype. However, these HPV vaccines were not therapeutic but prophylactic to cervical cancer and had dissatisfied effects on married women. Moreover, these vaccines were not only expensive but also scarce for Chinese women. Meanwhile, those vaccines were introduced to Chinese women lately, result in unclear long term effects and even may not fit them very well. Therefore, the HPV infection of Chinese women should not be neglected still.

The cervical cancer susceptibility can be affected mainly by HPV infection and varied with some other factors, such as HPV awareness and toll-like receptor gene polymorphisms (7–9). It was reported that due to the nation, socioeconomic status, age, ethnicity, and geographic location of populations were different, the HPV prevalence was substantially varied with different populations (10, 11), and the HPV genotype distribution also differed in various regions worldwide. Therefore, prevention and control measures for HPV infections should be implemented according to their local situation (6). Understanding the ecological diversity of HPV prevalence and its genotype distribution among various populations in different geographical regions of China is essential to optimize HPV vaccination and maximize its effects on Chinese women.

Hengyang city has 7.337 million permanent residents from over 40 nationalities and is the deputy center of Hunan province in China, which also is the second-largest city in this province. Therefore, the HPV prevalence and HPV genotype distribution of this city are significant for Chinese epidemiological data, and it can provide important advice for Chinese public health measures on cervical cancer prevention.

## Data Sources

In this study, the data of 12,053 women from the tertiary hospitals in Hengyang city between July 2018 and July 2019 were retrospectively analyzed. HPV testing was used as a routine examination for those suspected gynecologic patients from better-off families. HPV positive was identified by PCR, and HPV PCR-Flow fluorescence assay was used for HPV genotyping. 27 HPV genotypes were tested, including 17 HR-HPV genotypes (HPV 16, 18, 31, 33, 35, 39, 45, 51, 52, 56, 58, 59, 66, 68, 26, 53, and 82) and 10 LR-HPV genotypes (HPV 6, 11, 40, 42, 43, 44, 55, 61, 81, and 83).

### Statistical Analysis

The prevalence of HPV infection, presence of single or multiple HPV infections, and HPV genotypes, as well as, their corresponding 95% CIs were estimated with binomial distribution analysis. The Chi-square test was performed to compare the differences in the infection rates (the positive amount occupied among its corresponding whole population) of HPV or HR-HPV and the ratio-in-positive (the positive amount occupied among its corresponding positive population) of HR-HPV or LR-HPV among various patients. A *p*-value of 0.05 was considered statistically significant. The statistical data analysis was performed by SPSS version 13.0 (SPSS, IBM, USA).

### HPV Prevalence Among 12,053 Women

**Figure 1:**
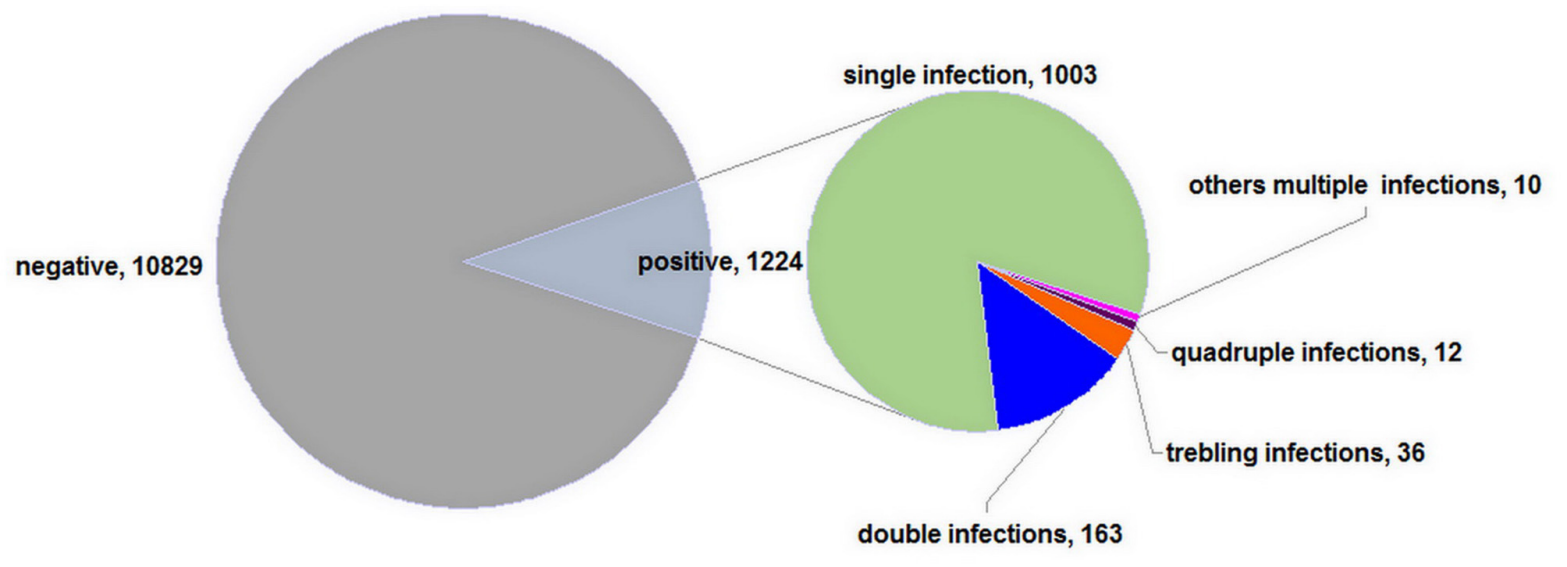
Human papillomavirus (HPV) prevalence among 12,053 women.

A total of 1,224 women were positive for HPV infection in 12,053 women, among which 1,003 were positive for a single genotype infection and 221 for multiple infections (Figure 1). It was reported that HPV prevalence in women worldwide had significant regional variations too, for example in 2012 Africa had the highest HPV prevalence (24%), followed by Eastern Europe (21%), Latin American (16%), and South-Eastern Asia (14%) in turn (12). In this study, the HPV infection rate was about 10.16% (1,224/12,053) overall, which was comparable with those reported in Zhejiang Province (13.3%) (13) in China, and those reported in other countries, such as in Guatemala (13%) (14) and in Iran, its neighboring countries, and Persian Gulf area (0.62–25%) (15), but was lower than those reported in some regions of China, such as Shenzhen City (15.9%) (16), the northwestern Yunnan Province (18.4%) (17), southern Yunnan Province (14.7%) (18), Yangcheng County (14.8%) (19), urban Tianjin (14.71%) (20), Shenyang City (16.8%) (21), south Taiwan (19.3%) (22), and those reported in other countries, such as Denmark, Norway, and Sweden in 2006–2008 (36.5%), and in 2012–2013 (34.5%) (23). The different HPV prevalence from Hengyang populations may be because that these various investigated people were from different geographical regions, from different nations, and with different ethnicity, lifestyle, and urbanization.

In the current study, 221 women were positive for multiple infections, among which a total of 163 patients were with double infections, 36 patients with triple infections, and 12 patients with quadruple infections, and there were patients even with septuple or sextuple infections (Figure 1). As shown in Figure 1, the multiple-genotype infection rate was 1.83% (221/12,053), which was similar to that in Beijing (1.5%) from 2014 to 2015 (24), but lower than those in Yunnan Province (4.2%) (17, 25). The high multiple-infection rate maybe because the diseases were more complicated or the illness more serious, which was in accordance with the report that multiple infections could elevate the risk of cervical cancer (26). Therefore, routine screening for HPV is very necessary for the health of women.

### Genotype Distribution Among 1,224 HPV Positive Women

**Figure 2:**
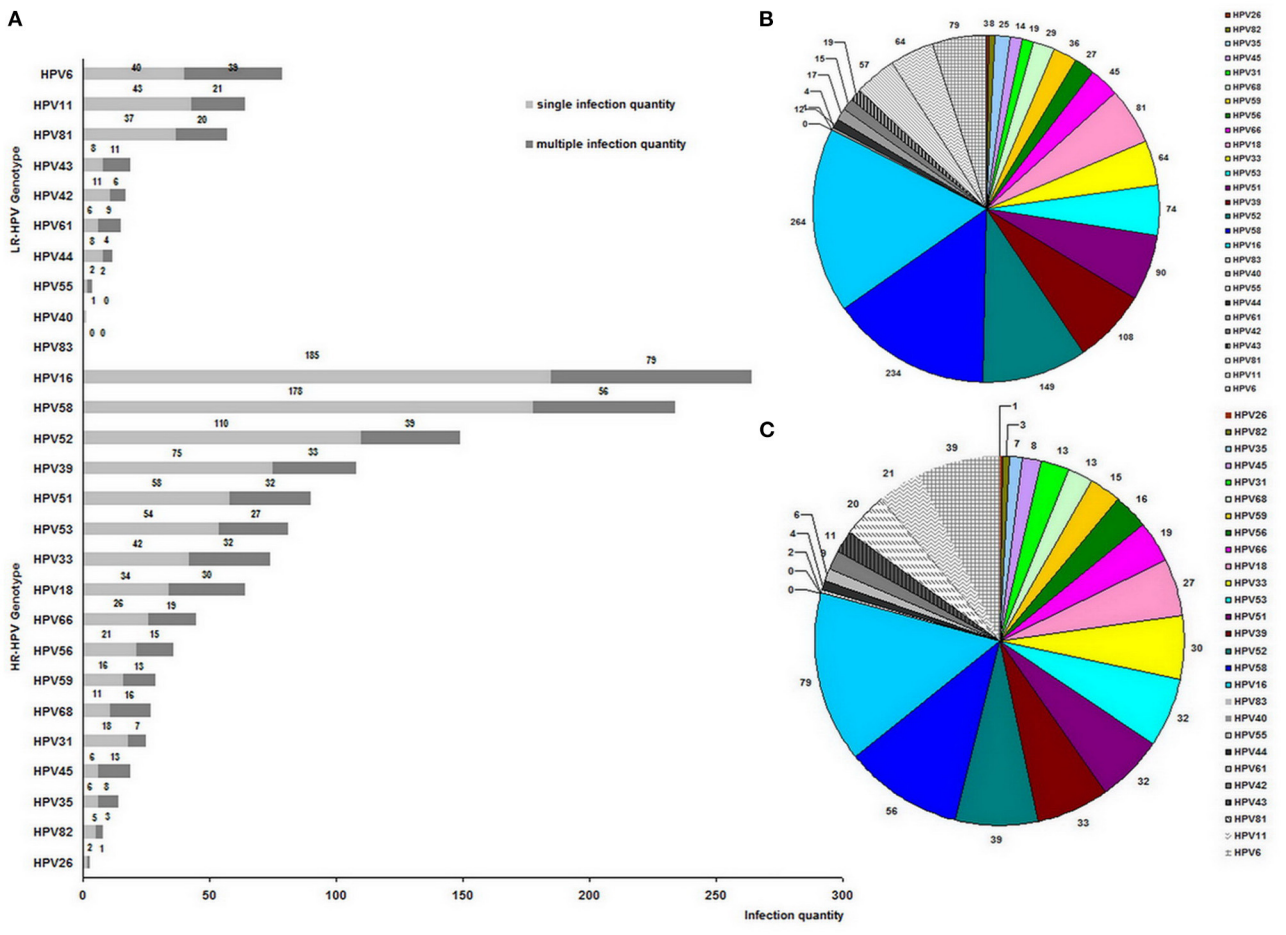
Genotype distribution of 1,538 clinical HPV strains among 1,224 HPV-positive women **(A)**, and genotype proportion of 1,538 clinical HPV strains **(B)**, and 535 clinical HPV strains from multiple infections **(C)**.

From 12,053 patients, 27 different HPV genotypes were identified. As shown in Figure 2, 1,027 women were infected by at least one type of HR-HPV, that is, HR-HPV infection rate was 8.52% (1,027/12,053), which was also a little lower than that found among women of Sichuan province (12.6%) (27), those among Han women from Mojiang county of Yunnan Province (12.6%) (28), and among the rural and urban women of southern Yunnan Province in China (11.4%) (17), and it was comparable with those reported from Beijing City (9.9%) (29), Tibet City (7.0%) (30), or Zhejiang Province (10.2%) (13) in China.

The distribution of the remaining top five genotypes was as follows in descending order: HPV58, HPV52, HPV39, HPV51, and HPV53, which differed from HPV prevalence in Guatemala, where the top six genotypes were HPV16, HPV18, HPV39, HPV58, HPV52, and HPV45 (14). Furthermore, this result about HPV genotype distribution was also different in that HPV52 had the most frequency followed by HPV39 and HPV68 among the urban population of Yunnan Province and some previous reports in China or other Asian countries (31, 32). However, this result was mainly consistent with the investigation in the Wuhan City of Hubei Province, where the most common genotype was HPV 52 followed by HPV 16, 58, 39, and 51 (33), and in agreement with the results in the rural population of Yunnan Province (18) and in Shanxi Province of China (34), where the relatively high prevalence of HPV16, HPV58, and HPV52 infections were reported, confirming that HPV58 and HPV52 were also responsible for HPV infections in Asia.

Among 12,053 women, HPV16 was the most frequent genotype with an infection rate of 2.19% (264/12,053). Moreover, the HPV16 positive patients occupied 21.57% among 1,224 HPV positive patients, that is, the ratio-in-positive of HPV16 was 21.57% (264/1,224), which was near to that (22%) in Guatemala (14), indicating that HPV16 was the most frequent as the general in all continents of the world. However, it could be seen that a slightly inferior ratio-in-positive (19.12%) was from HPV58, suggesting that HPV58 was the same major infected-genotype as HPV16 in China. In the Western country or South American continents, such as in Guatemala (14), HPV18 occupied the second frequency, but in Asian countries, HPV58 and HPV52 were more prevalent than HPV18 (35, 36). The variation of genotype distribution in Asia may be due to the different geographic regions, the diverse gene interactions, and various immune levels of patients (35). Therefore, the introduced HPV vaccine from the West does not fit Chinese women very well, suggesting that the study on the native therapeutic vaccine is still important for Chinese researchers.

The amount of all clinical HPV strains found from 1,224 positive women was 1,538, among which 535 clinical HPV strains were from 221 women with multiple infections. As shown in Figure 2B, HR-HPVs occupied 82.57% (1,270/1,538) and were more popular than exclusive LR-HPVs (17.42%, 268/1,538), which was the same as among some population from Yunnan Province (17, 18), Shanxi Province (19), Taiwan Province (22) in China and some Amerindians (37). Moreover, the infections by HR-HPV were more popular than those by exclusively LR-HPV among these women as Amerindians (37). Also, the cervical abnormalities were consistent with their high HPV infection prevalence. Furthermore, among the multiple infection genotypes, HPV16 was detected most frequently, and HR-HPVs were more popular than LR-HPVs (Figure 2C).

### Group Distribution Among 12,053 Patients

**Figure 3:**
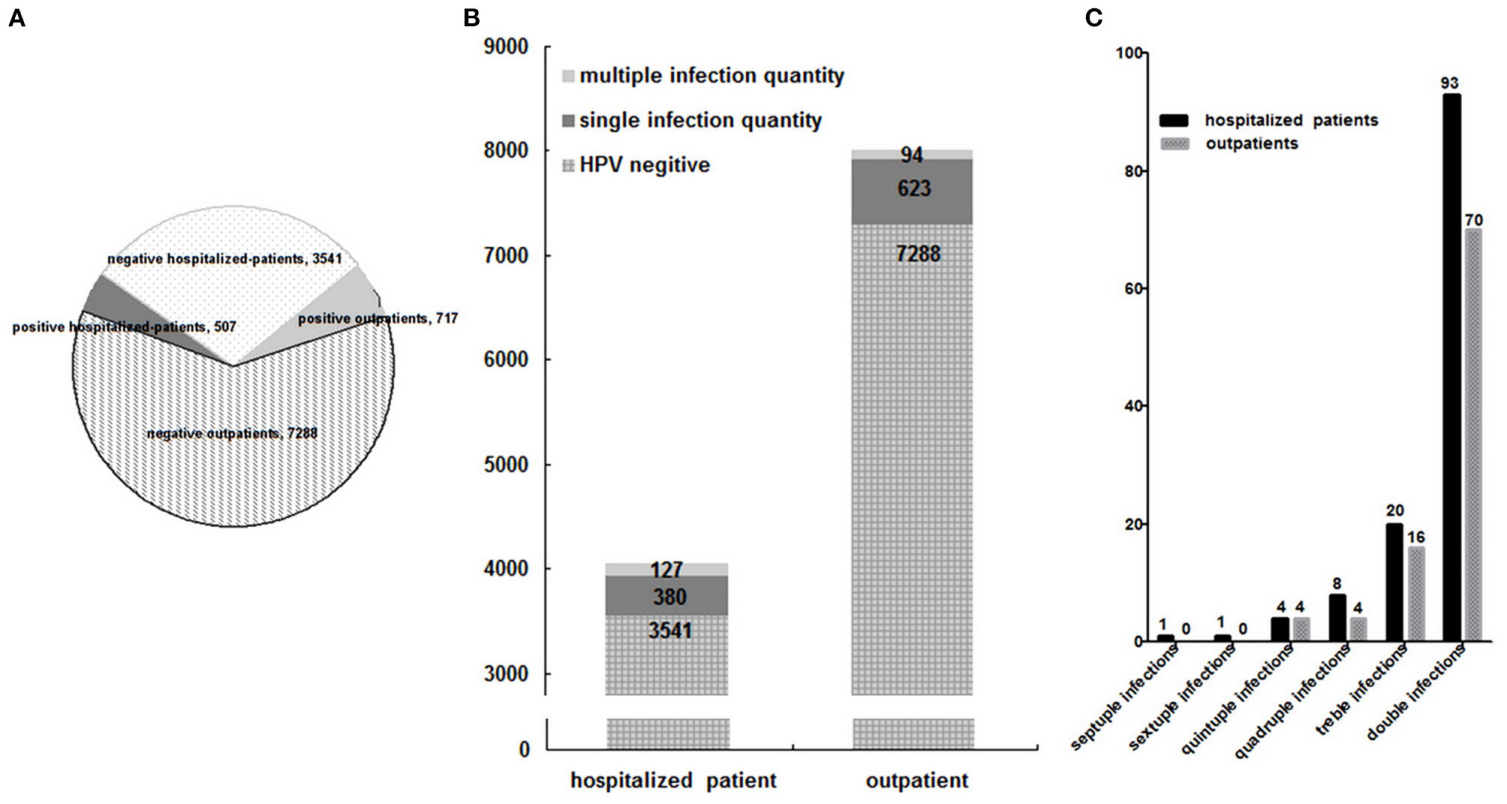
Group distribution of HPV infection among 12,053 patients **(A)**, and of multiple HPV infections among 12,053 patients **(B)**, and between outpatients and hospitalized-patients **(C)**.

Although the proportion of outpatients was 66.42% (8,005/12,053) more than that (33.58%, 4,048/12,053) of hospitalized-patients, their HPV infection rate (8.96%, 717/8,005) was lower than that (12.52%, 507/4,048) of hospitalized-patients (*p* < 0.01) (Figure 3A), the same as their multiple HPV infection rate (1.17%, 94/8,005; 3.14%, 127/4,048) (Figure 3B). It may be due to the fact that the hospitalized patients had severe or more complicated illnesses than outpatients, which may be explained by the fact that HPV infection, especially multiple HPV infections, would worsen some gynecologic related diseases or cause some severe diseases that occurred while suggesting that it was urgent to develop the therapeutic vaccine for HPV infection.

The difference of multiple-infection distribution between the outpatients and hospitalized-patients was not statistically significant (*p* > 0.05) (Figure 3C).

### Age Distribution of HPV Prevalence

As shown in Table 1, the ages of 12,053 patients were focused are from 21 to 50 years, which occupied 93.19% (11,232/12,053), and HPV-positive patients from 21 to 50 years correspondingly occupied 85.13% (1,042/1,224). Furthermore, the HPV infection rates of patients from above 60 years old (26.32%, 50/190) and below 21 years old (23.21%,13/56) were the most and second highest (*p* < 0.01), respectively, followed by that of 50–60 years group (20.70%, 119/575). The HPV infection rates of the most and the second prevalent group were slightly higher than those in Guatemala (younger women <30 years, corresponding to 22% and older women ≥60 years, corresponding to 15%) (14). Therefore, this age-specific prevalence curve did not well agree with that conducted in highly developed countries (38). The HPV infection rate of the youngest group (below 21 years) was high and different from that of Xishuang Banna (17), but in line with the data reported in some previous studies (30), suggesting that the higher urbanization may make the young women exposed to HPV infection more easily, which to a large extent may be ascribed to the early sexual activity. Moreover, it indicated that the HPV infection incidence showed a greater trend in the younger population and should not be neglected, indicating that the young people should improve their HPV-related knowledge more deeply for the prevention and intervention for cervical cancer (39).

**Table 1:** HPV prevalence of different age groups among 12,053 women.

**Age groups**	**HPV positive**		**HPV negative**	**Total**
	**HR**	**LR**		
Below 21	9	4	43	56
21–30	390	77	4,679	5,146
31–40	243	49	3,221	3,513
41–50	242	41	2,290	2,573
51–60	100	19	456	575
Above 60	43	7	140	190
Total	1,027	197	10,829	12,053

Among all HPV infections, HR-HPV infection was identified as the infection with at least one HR-HPV genotype, and its infection rate was 8.52% (1,027/12,053). The group above 60 years old with the highest HPV infection rate (26.32%, 50/190) had the highest HR-HPV infection rate (22.63%, 43/190), which was obviously higher than that (7.4%) in Beijing from 2014 to 2015. It may be due to that the investigated population in Hengyang was from gynecologic patients and was different from the population that received free screening in Beijing (24). On the contrary, the 50–60 years group had the third most infection rate in HPV (20.70%, 119/575) but the lowest in HR-HPV (17.39%, 100/575) (*p* < 0.01).

**Figure 4:**
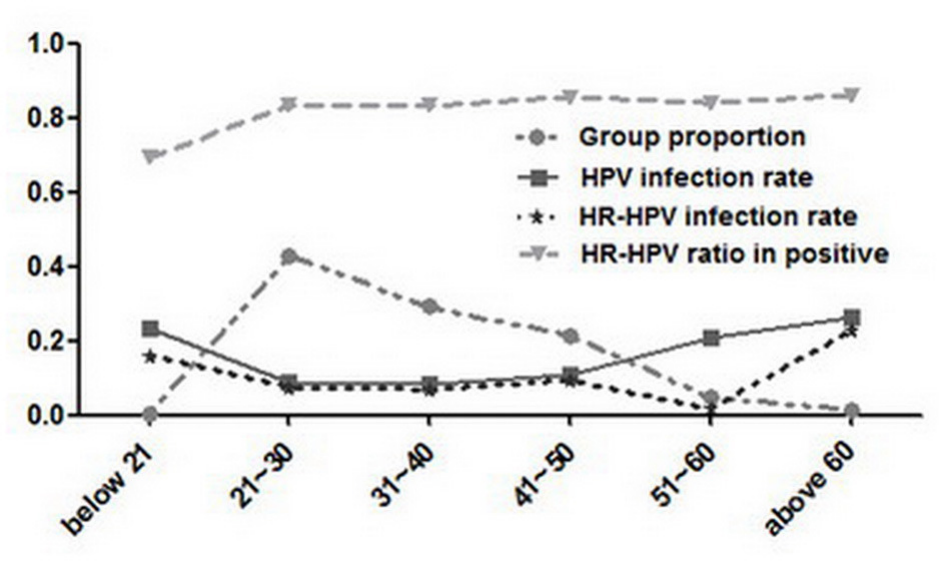
HPV prevalence of different age groups among 12,053 women.

In this study, the ratio-in-positive of HR-HPV (83.91%, 1,027/1,224) was higher than that of LR-HPV (16.09%, 197/1,224) among 1,224 HPV-positive patients. Although HR-HPV ratio-in-positive of the group above 60 years old (86%, 43/50) and below 20 years group (69.23%, 9/13) were the most or least, respectively, the difference between them (*p* > 0.05) or among those of all age groups (*p* > 0.05) had no statistical significance (Figure 4).

### HPV Prevalence Among Hospitalized-Patients

As seen from Table 2, among 4,048 hospitalized-patients, 507 patients were detected with HPV infections, among which 421 patients were infected with HR-HPVs and the other with only LR-HPVs. In the hospitalized-patients, the infection rate (10.40%, 421/4,048) and ratio-in-positive (83.04%, 421/507) of HR-HPV was more higher than those of only LR-HPV infection (2.12%, 86/4,048; 16.96%, 86/507), respectively. The difference of HR-HPV ratio-in-positive between the hospitalized-patients (83.04%, 421/507) and outpatients (84.52%, 606/717) was not statistically significant (*p* > 0.05). However, the HR-HPV infection rate (10.40%, 421/4,048) of the hospitalized-patients was higher than that of the outpatients (7.57%, 606/8,005) (*p* < 0.01) and even the average level (8.52%, 1,027/12,053), showing that the diseases related with HR-HPV infection would be more severe than those infected by LR-HPV only, thereby indicating the importance of the therapeutic vaccine.

**Table 2:** HPV prevalence of different age groups among 4,048 hospitalized-patients.

**Age groups**	**HPV positive**		**HPV negative**	**Total**
	**HR**	**LR**		
Below 21	4	1	16	21
21–30	137	29	1,690	1,856
31–40	94	21	1,075	1,190
41–50	116	21	615	752
51–60	51	12	87	150
Above 60	19	2	58	79
Total	421	86	3,541	4,048

The investigated population focused was 20–50 years old (93.19%, 11,232/12,053), so the same as the hospitalized-patients (93.82%, 3,798/4,048). Moreover, the patients of this age bracket were the most (82.45%, 418/507) among the 507 HPV-infected hospitalized-patients. Among 4,048 hospitalized-patients, the HPV infection rate (42%, 63/150) and HR-HPV infection rate (34%, 51/150) of 50–60 years patients were both the most (*p* < 0.01, *p* < 0.01), followed by those of above 60 years patients (26.58%, 21/79; 24.05%, 19/79). The high prevalence of HPV and HR-HPV among above 50 patients was in accordance with the situation that the cervical cancers are likely to happen in 50–70 years old patients, and might be attributable to their decreased immunity for clearing HPV infections, suggesting that the older people were the high-risk population for HPV.

**Figure 5:**
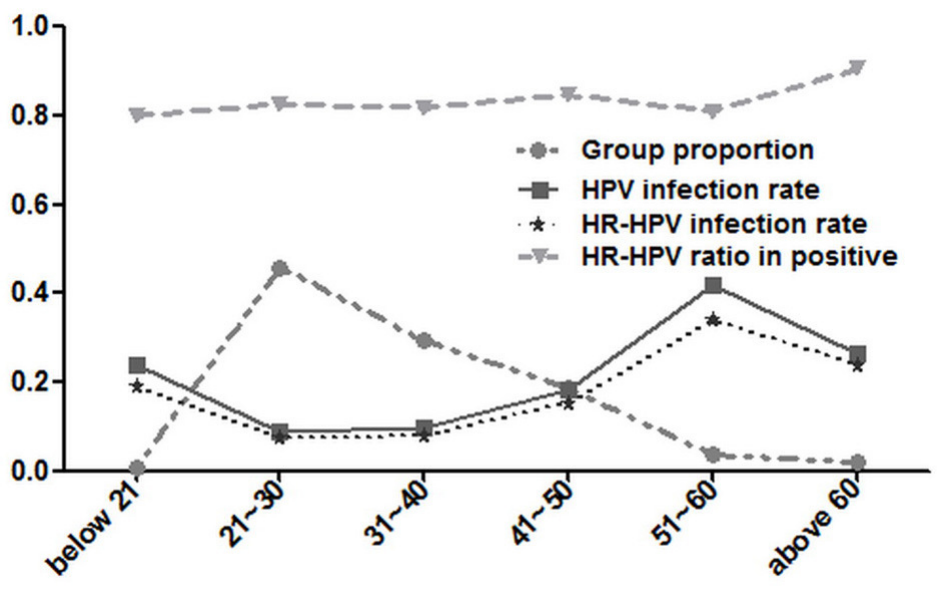
HPV prevalence of different age groups among 4,048 hospitalized-patients.

Among 4,048 hospitalized-patients, although the patients above 60 years old had the highest HR-HPV ratio-in-positive (90.48%, 19/21) and those below 21 years had the least (80%, 4/5), they had an obvious difference in HR-HPV ratio-in-positive (*p* > 0.05), and that difference among all age groups had no statistical significance (*p* > 0.05) (Figure 5).

### Pathomorphism Distribution Among 507 HPV-Infected Hospitalized-Patients

Among 507 hospitalized-patients with HPV infection, 284 patients had been performed with histopathologic examination according to their illness, but the other 223 patients did not need examination. Among the 284 HPV-infected hospitalized-patients with the histopathologic examination, 70 patients were with cervical carcinoma or its precancerous lesions, 157 with uterine fibroid or its related lesions, and 57 with the pelvic neoplasm or its related lesions. Among 507 HPV-infected hospitalized-patients, the HR-HPV ratio-in-positive of 284 patients with the histopathologic examination was 90.49% (257/284) and higher than that (78.03%, 174/223) of 223 patients without (*p* < 0.01) or even the average level (83.04%, 421/507) (*p* < 0.05), which confirmed that HR-HPV can make the disease severe and even result in organ damage.

**Figure 6:**
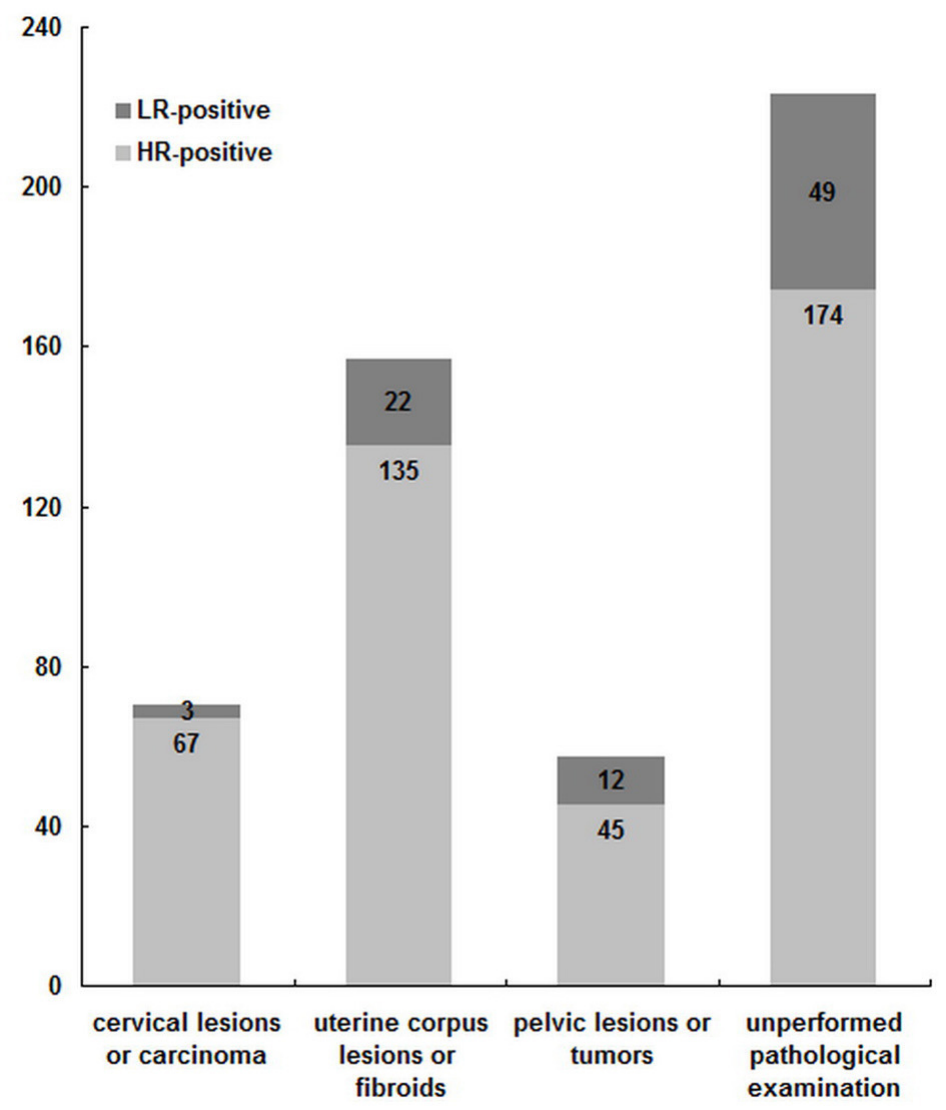
Pathomorphism distribution among 507 HPV positive hospitalized-patients.

Among 284 HPV-infected hospitalized-patients with the histopathologic examination, the patients with cervical carcinoma or its precancerous lesions had the highest HR-HPV ratio-in-positive (95.71%, 67/70) (*p* < 0.05) (Figure 6), suggesting that cervical carcinoma or its precancerous lesions had a close relationship with HR-HPV virus.

**Figure 7:**
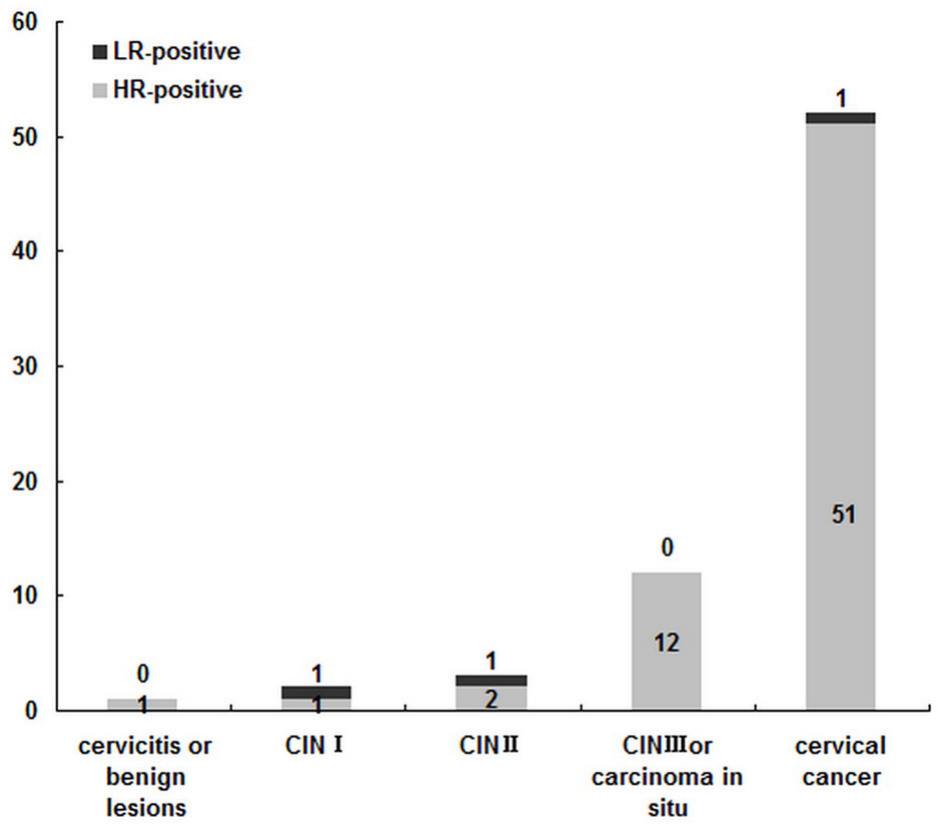
HPV prevalence among 70 HPV positive patients with cervical lesions or carcinoma.

As shown in Figure 6, among 70 HPV infected patients with cervical lesions or carcinoma, 52 patients were with cervical squamous carcinoma, and HR-HPV infection occupied 98.57% (69/70). Among the above 52 patients, the other top six genotypes were HPV52, HPV39, HPV51, HPV58, HPV18, and HPV53 (Figure 7), which was mainly consistent with the frequency of HPV16, HPV58, HPV52, HPV39, HPV51, and HPV53 infection in the whole population, and this was similar with that in Beijing from 2014 to 2015 (24). It was worth mentioning that there was one patient with HPV6 single infection among cervical carcinoma patients. Among the above patients, the HPV16 ratio-in-positive (61.54%) was comparable to that in Beijing from 2014 to 2015 (58.54%) (24) and was obviously more than that among esophageal carcinoma samples (45.24%) (40). Moreover, HPV16 infections occupied 61.54% (32/52) among cervical cancer samples, indicating that the HPV16 virus had played a key role in the development of cervical squamous carcinoma. It was worth mentioning that in this current study there was one cervical carcinoma patient with HPV 6 single infection, suggesting that the role of LR-HPV in cervical squamous carcinoma could not be neglected.

## Conclusion

On the basis of the study on the awareness and knowledge levels on HPV (41), this study demonstrated that HPV prevalence and its genotype distribution in Hengyang city of Hunan province in China were mainly in accordance with the present situation of HPV prevalence in China. The controlled variations may occur due to the different populations in different socioeconomic and ethnic environments with different lifestyles and living standards from the different geographical regions. This study indicated that the HPV prevention and control strategies need to be carried out urgently for Hengyang city of Hunan in China, and it also highlighted the importance of HPV53 and HPV39 in China, which can guide the further study for the next generation of HPV vaccines.

## Data Availability Statement

The original contributions presented in the study are included in the article/supplementary material, further inquiries can be directed to the corresponding author/s.

## Ethics Statement

The human data in present study were collected in line with the Helsinki Declaration and was approved by the Ethical Review Committees of University of South China.

## Author Contributions

SYT analyzed and interpreted the patient data and was a major contributor in writing the manuscript. YQL, YH, and HYS also analyzed and interpreted the patient data collaboratively. YPW and YMW performed the collection of data. All authors read and approved the final manuscript.

## Funding

The authors acknowledge the grants of the Provincial Natural Science Foundation of Hunan Province (2021JJ50014), Scientific Research Project of Hunan Provincial Health Commission (202105012137), Municipal Science project of Hengyang City in Hunan Province (202002042122), National Natural Science Foundation of China (81402169), Educational Committee Foundation of Hunan Province (19C1616), Hunan Provincial Key Laboratory for Special Pathogens Prevention and Control (2014-5) and Construct Program of the Double First-Class discipline of University of South China (2020SYL).

## Conflict of Interest

The authors declare that the research was conducted in the absence of any commercial or financial relationships that could be construed as a potential conflict of interest.

## Publisher's Note

All claims expressed in this article are solely those of the authors and do not necessarily represent those of their affiliated organizations, or those of the publisher, the editors and the reviewers. Any product that may be evaluated in this article, or claim that may be made by its manufacturer, is not guaranteed or endorsed by the publisher.
